# The genomic basis of adaptation to the fitness cost of rifampicin resistance in *Pseudomonas aeruginosa*

**DOI:** 10.1098/rspb.2015.2452

**Published:** 2016-01-13

**Authors:** Qin Qi, Macarena Toll-Riera, Karl Heilbron, Gail M. Preston, R. Craig MacLean

**Affiliations:** 1 Department of Zoology, University of Oxford, Oxford, UK; 2 Department of Plant Sciences, University of Oxford, Oxford, UK; 3 Institute of Science and Technology Austria, Klosterneuburg, Austria; 4 Institute of Evolutionary Biology and Environmental Studies, University of Zurich, Zurich, Switzerland

**Keywords:** experimental evolution, whole-genome sequencing, rifampicin resistance, compensatory adaptation, general adaptation

## Abstract

Antibiotic resistance carries a fitness cost that must be overcome in order for resistance to persist over the long term. Compensatory mutations that recover the functional defects associated with resistance mutations have been argued to play a key role in overcoming the cost of resistance, but compensatory mutations are expected to be rare relative to generally beneficial mutations that increase fitness, irrespective of antibiotic resistance. Given this asymmetry, population genetics theory predicts that populations should adapt by compensatory mutations when the cost of resistance is large, whereas generally beneficial mutations should drive adaptation when the cost of resistance is small. We tested this prediction by determining the genomic mechanisms underpinning adaptation to antibiotic-free conditions in populations of the pathogenic bacterium *Pseudomonas aeruginosa* that carry costly antibiotic resistance mutations. Whole-genome sequencing revealed that populations founded by high-cost rifampicin-resistant mutants adapted via compensatory mutations in three genes of the RNA polymerase core enzyme, whereas populations founded by low-cost mutants adapted by generally beneficial mutations, predominantly in the quorum-sensing transcriptional regulator gene *lasR*. Even though the importance of compensatory evolution in maintaining resistance has been widely recognized, our study shows that the roles of general adaptation in maintaining resistance should not be underestimated and highlights the need to understand how selection at other sites in the genome influences the dynamics of resistance alleles in clinical settings.

## 1. Introduction

The evolution of antibiotic resistance in pathogenic bacteria is typically accompanied by fitness costs that are expressed in terms of reduced growth rate, competitive ability and virulence [1–3]. Fitness costs generate selection against resistance when pathogen populations encounter antibiotic-free environments, as occurs during transmission between hosts or when antibiotic use is discontinued. Because exposure to high doses of antibiotic is transient, resistance will only be maintained in the long term if resistant populations can evolve adaptations that offset the cost of resistance. Therefore, understanding the mechanisms that allow resistant bacterial populations to evolve increased fitness is of fundamental importance to understanding the long-term maintenance of resistance in pathogenic bacteria.

One possible mechanism through which bacteria can overcome the cost of resistance is by acquiring compensatory mutations that reduce or eliminate the fitness costs associated with resistance alleles by recovering the functional defects associated with resistance mutations [4–9]. Alternatively, it is possible for antibiotic-resistant populations to overcome the fitness cost of resistance by acquiring generally beneficial mutations that increase fitness without offsetting the cost of resistance *per se*. Unlike compensatory mutations, generally beneficial mutations contribute to an increase in fitness, regardless of the genetic background in which they arise [10,11]. In this scenario, resistant populations overcome the cost of resistance by adapting to general environmental conditions, such as nutrient availability and the presence of stressors. The dominant view that has emerged from studies of the long-term evolution of antibiotic-resistant populations is that adaptation to the cost of resistance is driven by compensatory mutations [1,2,12–14]. Compensatory mutations have been identified in clinical pathogen populations, especially *Mycobacterium tuberculosis* [15–17], and compensatory adaptation has emerged as a central explanation for the long-term maintenance of resistance in pathogen populations [3].

Although compensatory mutations have been identified in a wide range of systems, the fact that resistant populations can also evolve by generally beneficial mutations that increase fitness, irrespective of antibiotic resistance, is far less frequently mentioned in current discussions of antibiotic resistance evolution. It has been estimated that there are, on average, 12 possible compensatory mutations per deleterious mutation in bacteria [18], and the rate of compensatory mutation should therefore be in the order of 12 sites per genome x 9 × 10^−11^ mutations per site per generation under laboratory conditions [19], which corresponds to ≈1 × 10^−9^ per genome per generation. On the other hand, generally beneficial mutations have been estimated to occur at a rate between 1 × 10^−8^ and 1 × 10^−5^ per genome per generation under laboratory conditions, which is 10–10 000 times higher than our crude estimate of the rate of compensatory mutation [20–23]. This asymmetry suggests that, all else being equal, generally beneficial mutations are expected to contribute more towards adaptation in antibiotic-resistant populations than compensatory mutations do.

However, adaptation in asexual populations is driven by a small minority of mutations with relatively large benefits that overcome stochastic drift and competition from rival beneficial mutations owing to clonal interference [20,22,24,25]. Compensatory mutations are therefore expected to make a disproportionately large contribution to adaptation when they are associated with large fitness benefits relative to generally beneficial mutations. Because compensatory adaptation directly recovers the functional defects associated with resistance mutations [5,7,8,26], compensatory mutations should confer large fitness benefits in populations carrying very costly resistance mutations. However, the fitness benefits associated with compensatory mutations are expected to be small in populations carrying low-cost mutations. This population genetic framework predicts that the likelihood of adaptation by compensatory mutations should increase with the cost of resistance [27]. Crucially, studies of evolution in antibiotic-resistant populations have largely focused on adaptation in populations that carry very costly resistance mutations [6,28–32]. We postulate that the roles played by general adaptation in eliminating the fitness cost of antibiotic resistance are greater than currently thought.

To test the hypothesis that high fitness costs promote evolution by compensatory adaptation, we allowed populations of eight isogenic rifampicin-resistant isolates of the opportunistic pathogen *Pseudomonas aeruginosa* that carry different fitness costs, as well as their rifampicin-sensitive ancestor, to evolve for 300 generations in an antibiotic-free rich medium. Extensive whole-genome sequencing was used to systematically identify compensatory mutations and generally beneficial mutations in evolved endpoint populations.

## 2. Results and discussion

### (a) Evolved rifampicin-resistant mutants recover from the fitness cost of resistance

**Figure 1.**
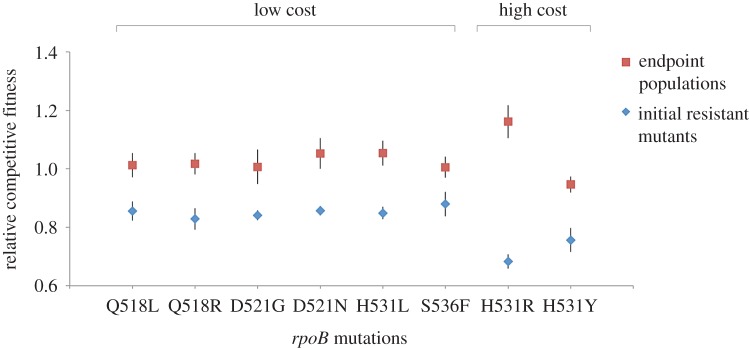
Adaptation to the cost of rifampicin resistance. This figure shows the competitive fitness (mean ± s.e; *n* = 3) of the eight *rpoB* mutants used to found the selection experiments (blue diamonds) and the average fitness of three endpoint populations (red squares) evolved from each *rpoB* mutant. The *rpoB* mutations carried by the rifampicin-resistant mutants are specified in the names assigned to the mutants. The relative fitness of all endpoint populations converged towards that of the rifampicin-sensitive ancestral strain (standardized to a value of 1). In all cases, the average relative fitness of the endpoint populations showed a significant increase compared with that of the initial *rpoB* mutant (two-sample *t*-test, *p* < 0.05).

To study adaptation to the cost of rifampicin resistance, we allowed three independently propagated populations of eight different rifampicin-resistant *rpoB* mutants that carried different fitness costs to evolve in a rifampicin-free culture medium for 300 generations. Fitness varied considerably between the rifampicin-resistant mutants that were used to initiate the selection experiment (figure 1: one-way ANOVA, *F*_7,16_ = 4.66, *p* = 0.0052). Fitness increased over the course of the selection experiment (paired *t*-test, *t*_7_ = 5.47, *p* = 0.0009), and the fitness of the evolved populations was very similar to that of the rifampicin-sensitive ancestral strain that the resistant mutants were evolved from. These results clearly demonstrate that selection in the absence of antibiotics can rapidly eliminate the substantial fitness cost that is associated with rifampicin resistance for a range of *rpoB* mutations.

### (b) Testing for compensatory adaptation using whole-genome sequencing

To determine the genetic basis of adaptation in antibiotic-resistant populations, we performed whole-genome sequencing on three randomly selected colonies isolated from each evolved endpoint population (electronic supplementary material, table S1: 8 mutants × 3 populations per mutant × 3 isolates per population = 72 isolates). As a control experiment, we also sequenced the genomes of evolved isolates from rifampicin-sensitive ancestral populations that were evolved for the same number of generations (electronic supplementary material, table S2: 12 populations × 3 isolates per population = 36 isolates). Non-synonymous single-nucleotide polymorphisms (SNPs) and short indel (insertion and deletion) mutations of less than 1 kb in protein-coding regions were the most common forms of mutations that we identified. Silent and intergenic mutations were found at low frequencies, as were large deletions and duplications spanning more than one gene.

**Figure 2.**
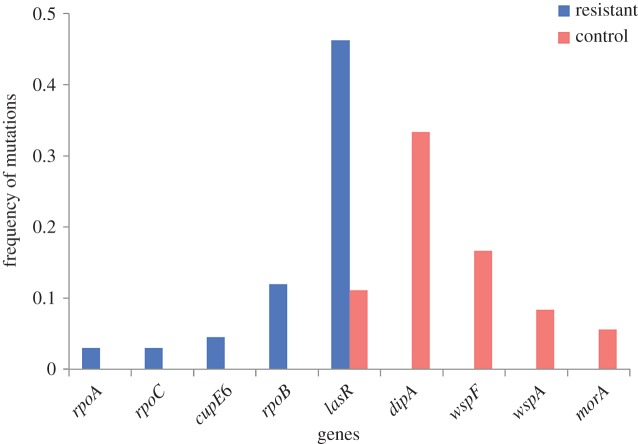
Targets of selection. In the frequency distribution of genes that acquired mutation(s) in at least two independently evolved endpoint populations, a high degree of parallel evolution can be observed in both the rifampicin-resistant and control populations. At the same time, a distinct difference in the spectrum of genes that were most frequently mutated can also be observed.

The mutations that we identified may include beneficial, neutral or mildly deleterious mutations. Parallel evolution provides a hallmark of genes that are under strong positive selection [33–36], and we therefore focused our analysis on genes in which we detected parallel evolution. In our study, parallel evolution, in a particular gene, can be demonstrated either by mutations in different populations or by different mutations within the same endpoint population. Our results broadly indicate that such parallel evolution was very common in both the evolved resistant and control populations. Among the resistant mutant populations (figure 2), five genes contained 69% of all mutations that were detected in the sequenced endpoint isolates evolved from the rifampicin-resistant mutants (highlighted in blue), whereas five genes covered 75% of all mutations found in the evolved control populations (highlighted in red).

Because compensatory mutations interact epistatically with resistance mutations to recover fitness [26,31,37], we expected compensatory mutations to be found in evolved resistant populations, but not in rifampicin-sensitive ancestral populations. Previous studies have shown that second-site mutations in RNA polymerase subunits (*rpoA, rpoB* and *rpoC*) can compensate for the cost of rifampicin resistance [5,7,16,26]. Consistent with this, we found nine independently evolved point mutations in these RNA polymerase genes among the resistant populations, but no RNA polymerase mutations were detected in the evolved control populations. Interestingly, one of the *rpoB* mutations represents a reversion mutation that swept to fixation in a population that was initiated by the H531R mutant. The reversion mutation restored the rifampicin-sensitive phenotype, which constitutes an evolutionary reversal of antibiotic resistance. Several lines of evidence suggest that the remaining RNA polymerase mutations are compensatory mutations rather than generally beneficial mutations. First, the intragenic second-site mutations in *rpoB* found in this study (E528D, H531C and N573S) are known to recover the reductions in transcriptional efficiency and fitness costs associated with the original *rpoB* mutations [5]. Second, we observed three independent examples of parallel evolution in *rpoB* at an amino acid level. An additional mutation in the same codon as the original *rpoB* mutations H531R and H531Y can result in a common amino acid substitution H531C, which was observed in two populations founded by H531R and one population founded by H531Y (electronic supplementary material, table S1). H531C can offset the fitness cost of the original *rpoB* mutations without altering the rifampicin resistance phenotype [38].

**Figure 3.**
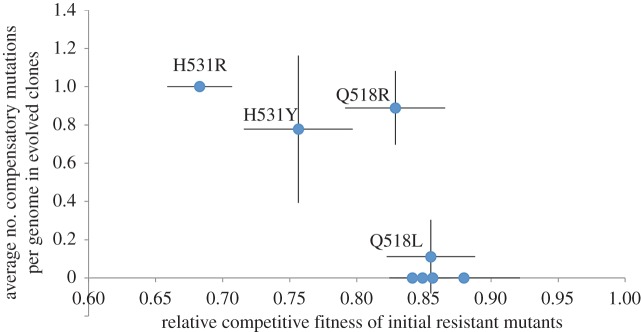
High fitness costs drive compensatory adaptation in rifampicin-resistant populations of *P. aeruginosa*. This graph shows the number of compensatory mutations in RNA polymerase per evolved isolate (mean ± s.e; *n* = 3) as a function of fitness cost associated with the eight rifampicin resistance mutations (mean ± s.e; *n* = 3). We included the reversion mutation in this analysis, because it is an adaptation to the cost of rifampicin resistance. The mean number of compensatory mutations per isolate increases with the cost of resistance, as judged by a Spearman's rank-order correlation (*p* < 0.05). (Online version in colour.)

To test the hypothesis that low fitness costs constrain compensatory adaptation, we tested for a negative correlation between the frequencies of RNA polymerase mutations and the initial cost associated with each *rpoB* mutation (figure 3). In support of our hypothesis, we found a significant negative correlation between the number of compensatory mutations per genome and the initial cost associated with resistance (Spearman's rank correlation: *ρ* = −0.786, *r*^2^ = 0.618, *F*_1,6_ = 9.72, *p* = 0.0206). Almost all isolates from populations that were founded by very costly resistance mutants, such as H531R and H531Y, carry compensatory mutations, whereas no compensatory mutations were found in populations that were founded by low-cost resistance mutations such as S536F.

### (c) Adaptation in antibiotic-resistant populations is dominated by generally beneficial mutations

Although we found evidence for compensatory adaptation when the cost of resistance was large, the dominant genetic mechanism of adaptation in antibiotic-resistant populations was mutations in *lasR*, a key transcriptional regulator gene involved in quorum-sensing [39,40]. About 71% of all endpoint populations founded by resistant mutants had at least one sequenced isolate with mutations in *lasR*. There was also a high diversity of mutations in *lasR*, which include 26 independently evolved SNPs and short indel mutations, as well as four independent large deletions (more than 2 kb) affecting *lasR* and adjacent genes. These mutations are thought to disrupt LasR function and result in quorum-sensing deficiency [41–45]. For example, the missense mutation A231V, which was observed in two independent populations founded by the *rpoB* mutant Q518R, is known to disrupt LasR functions and quorum-sensing-regulated phenotypic traits [41]. Mutations in *lasR* led to increased fitness in *rpoB* mutants (electronic supplementary material, figure S1). In agreement with this, numerous studies have shown that *P. aeruginosa* populations adapt to novel environments through the loss of LasR function [42–46].

*lasR* mutations were also detected in the evolved control populations (electronic supplementary material, table S2), confirming that *lasR* mutations were not compensatory. More specifically, three independent *lasR* mutations we observed are known to compromise LasR function by eliminating the start codon (M1I), deleting the *lasR* promoter region [47] or preventing LasR multimer formation via the P74L mutation [48]. However, the proportion of control populations that had at least one sequenced isolate with *lasR* mutations (33%) was lower compared with that of resistant populations (71%), so it remains to be understood why such discrepancies exist between the resistant and control populations.

### (d) The evolutionary dynamics of general and compensatory adaptation

**Figure 4.**
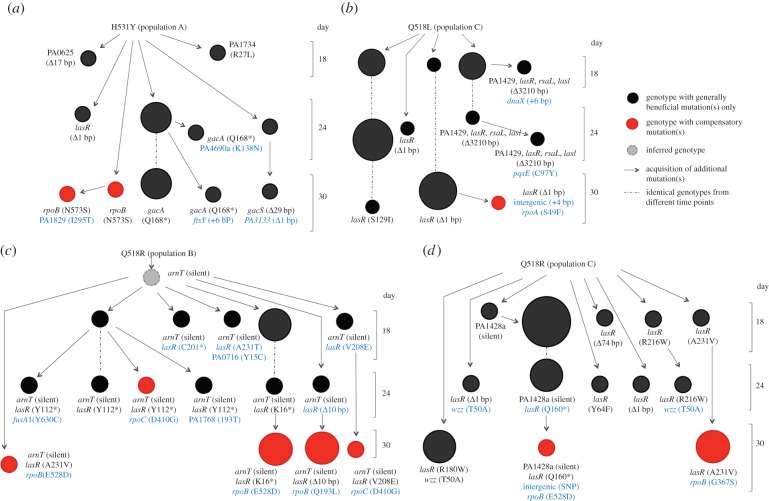
Clonal interference constrains compensatory adaptation. A schematic diagram representing the phylogeny inferred for six randomly selected isolates sampled from the 18th, 24th and 30th (final) days for four populations in which the co-occurrence of generally beneficial mutations and compensatory mutations was detected in the endpoint populations. Circles represent the observed genotypes, and the diameter of a circle indicates the frequency of that isolate in the population (*n* = 1, 2 or 3). Red circles denote isolates carrying compensatory mutations, while black circles denote isolates carrying generally beneficial mutations but not compensatory mutations. Each thin arrow represents the acquisition of one or more additional mutations with respect to the previous genotype. Mutations carried by individual isolates are shown in text, with newly acquired mutations in blue and previously acquired mutations in black.

To better understand the conflict between compensatory adaptation and general adaptation, we studied the dynamics of adaptation by sequencing a time series of isolates from a subset of our selection lines. We focused on populations in which both generally beneficial mutations and compensatory mutations were identified (electronic supplementary material, table S1). The co-occurrence of generally beneficial mutations and compensatory mutations implies that these populations are likely to provide direct insights into the outcome of competition between these two classes of beneficial mutations. Based on the sequencing results, we constructed the evolutionary history for these four populations (figure 4). The key insight that emerged from our phylogenetic inference was that general adaptation tends to precede compensatory adaptation. In three of the four populations, general adaptation initially occurred through the spread of *lasR* mutations during the first 24 days of the experiments (figure 4*b*–*d*). Successful *lasR* mutants then acquired compensatory mutations in RNA polymerase genes, which began to increase to detectable frequency by the end of the experiment. In the H531Y-A population (figure 4*a*), initial adaptation was dominated by the spread of mutations in the *gacA* gene. GacS/GacA modulates the expression of more than 500 genes of the RsmA regulon and is indirectly involved in the regulation of quorum-sensing [49–51]. Subsequently, isolates carrying compensatory mutations in the *rpoB* gene also began to increase to detectable frequency by day 30.

Several factors are likely to have favoured this repeatable evolutionary dynamic of general adaptation followed by compensatory adaptation. We detected a vast diversity of *lasR* mutations, suggesting that any mutation which disrupts LasR production and the quorum-sensing phenotype is beneficial under these experimental conditions. By contrast, we found a relatively small number of compensatory mutations in RNA polymerase, which supports the idea that compensatory mutations are rare [52]. This asymmetry is illustrated by the observation that the frequency of repeated independent mutations in *rpoB* (3/7) was much higher than that in *lasR* (1/30) among the resistant populations. *lasR* mutations also confer large fitness benefits. For example, three double mutants that carry single *lasR* mutations in addition to their original *rpoB* mutations, showed an increased fitness of between 15.6% and 32.3% (electronic supplementary material, figure S1). This suggests that the fitness benefit associated with *lasR* mutations was likely to be comparable to, or greater than, the benefit associated with compensatory mutations, except when the cost of resistance was very large.

Surprisingly, we found that the control populations often carried mutations in genes that were not mutated in resistant populations (figure 2), including *wspA* and *wspF* from the *wsp* operon (*n* = 9), *dipA* (*n* = 12), as well as *morA* (*n* = 2). The absence of *wspA/wspF*, *dipA* and *morA* mutations in the resistant populations raised the possibility that the fitness benefit conferred by these mutations could be contingent on the absence of *rpoB* mutations (sign epistasis) [53]. To test this hypothesis, we measured the fitness of isolates carrying *rpoB* mutations with *dipA* or *morA* mutations and found that *dipA* and *morA* mutations were beneficial in the presence of *rpoB* mutations [54], implying that sign epistasis cannot explain the absence of mutations in these genes in the evolved resistant isolates. It is conceivable that the different amounts of time spent by the various strains in the stationary phase could have contributed to these differences in the mutational spectrum.

## 3. Conclusion

The idea that compensatory adaptation eliminates the cost of resistance and allows resistance alleles to effectively persist in bacterial populations has been extensively studied in evolutionary models of antibiotic resistance [2,3,13,55–57]. Here, we examine the underlying genomic basis of adaptation in rifampicin-resistant populations of the pathogenic bacterium *P. aeruginosa*. Although rifampicin-resistant populations quickly evolved to overcome their initial fitness cost, compensatory mutations were fixed in populations founded only by highly costly resistant mutants. Importantly, many studies of evolution in antibiotic-resistant populations tend to focus on evolution in populations carrying very costly resistance mutations [6,28–32]. Our results suggest that the role of generally beneficial mutations in overcoming the fitness cost of resistance may be more important than currently thought. Given the functional diversity of antibiotic resistance mechanisms, more insight into the conflicts between these two types of mutations could be gleaned by performing experimental evolution studies using antibiotic-resistant mutants that carry other types of resistance mutations.

Although compensatory adaptation provides a more direct solution to the cost of resistance, two constraints are likely to restrict compensatory evolution. First, generally beneficial mutations are more common than compensatory mutations. While compensatory mutations usually occur in the same protein or pathway as antibiotic resistance mutations do [4,52], generally beneficial mutations are expected to occur at many different sites in the genome. In our study, this asymmetry is highlighted by the rarity of repeated independent mutations in genes involved in general adaptation, notably in *lasR*, versus the higher frequency of repeated nucleotide substitutions in *rpoB*, which was involved in compensatory adaptation. Second, generally beneficial mutations can have large effects on fitness that surpass those associated with compensatory mutations (electronic supplementary material, figure S1). We argue that compensatory adaptation tends to occur when the cost of resistance is large, because compensatory mutations provide very large fitness benefits in populations carrying costly resistance mutations.

Given that the spread of compensatory mutations can be delayed by the spread of generally beneficial mutations owing to clonal interference, selection for compensatory adaptation will be relatively ineffective under conditions where selection at other sites in the genome is strong. A number of important selective forces are likely to exert strong and recurrent selective pressure on populations of bacterial pathogens, such as antibiotic use [58,59], bacteriophage [60] and the immune system [61], suggesting that compensatory adaptation may be difficult to acquire in clinical settings. These constraints are probably especially important in opportunistic pathogens, because they are essentially invading a novel niche that imposes distinct selective pressures when they establish infections in human hosts [35,62,63]. In this respect, it is interesting to note that the best-characterized example of compensatory adaptation in a clinical setting, rifampicin resistance in *M. tuberculosis* [7,15,16], comes from an obligate pathogen that is well adapted to life in human hosts [64].

Whole-genome sequencing is now being used extensively to study the population biology of bacterial pathogens, and it is often thought that mutations which are found in antibiotic-resistant isolates are compensatory mutations [15,59,65–67]. Although there are some excellent examples of compensatory adaptation in clinical pathogens [6,15,16,68–70], it is also apparent that compensatory evolution is not ubiquitous [57]. Our study highlights some of the obstacles to evolution by compensatory mutations, but their consequences remain unclear. On the one hand, it is possible that rapid adaptation by generally beneficial mutations effectively eliminates selection against antibiotic-resistant pathogen lineages. In this scenario, resistance alleles continue to carry a cost, but selection against resistant lineages is offset by the beneficial mutations carried at other sites in the genome. Importantly, antibiotic use results in a large increase in the population size of antibiotic-resistant lineages, suggesting that they should enjoy an increased likelihood of acquiring generally beneficial mutations relative to sensitive strains that are suppressed by antibiotic use. Compensatory mutations interact epistatically with resistance mutations such that the loss of resistance mutations becomes deleterious once compensatory mutations have been acquired. By delaying the spread of compensatory mutations, it is possible that generally beneficial mutations increase the window of opportunity for reversion to antibiotic sensitivity. It is hoped that future work will shed light on the roles that selection for generally beneficial mutation plays in the maintenance of resistance.

## 4. Material and methods

### (a) Selection experiment

The eight rifampicin-resistant *rpoB* mutants used in the selection experiment were evolved from the rifampicin-sensitive PAO1::mini-Tn7-p*LAC*-*lux* ancestral strain using a fluctuation test [5]. All strains were streaked on M9KB agar plates [71] and incubated overnight at 30°C. On the next day, three independent colonies per *rpoB* mutant strain were randomly selected for propagating three independent populations (denoted A–C) during the selection experiment. About 1 ml of M9KB liquid culture medium [71] was inoculated with a colony in the absence of rifampicin. The bacterial cultures were incubated overnight with shaking (225 r.p.m.) at 30°C. On the next day, 1 µl of each overnight culture was diluted 1000-fold in fresh M9KB medium and incubated overnight under the same experimental conditions. The serial transfer was repeated for 30 consecutive days, or approximately 300 generations. Glycerol stocks of all populations were prepared on the 18th, 24th and 30th (final) days of the selection experiment. In a separate set of control experiments, 12 independent colonies of the rifampicin-sensitive ancestral strain were randomly selected for propagating 12 independent populations (denoted A–L) using the same experimental procedures as described above.

### (b) Competitive fitness assay

The competitive fitness of the endpoint populations of each of the propagated populations was quantified relative to the rifampicin-sensitive ancestral strain as previously described [5]. Briefly, overnight cultures of the endpoint populations, the PAO1::mini-Tn7-p*LAC*-*lux* ancestral strain and a GFP-tagged derivative strain of PAO1 were diluted 1 : 10 in M9KB medium and regrown to early exponential phase at 30°C with continuous shaking (225 r.p.m.). Each non-fluorescent strain was mixed with the GFP-tagged strain, diluted 200-fold in M9KB medium and incubated in Nunc 96-well microplates (Thermo Scientific, USA) at 30°C overnight for approximately 24 h with continuous shaking. The proportion of fluorescent and non-fluorescent cells in each co-culture was determined using an Accuri C6 flow cytometer (BD Biosciences, USA) before and after the incubation. The competitive fitness of each non-fluorescent strain was calculated as the ratio of population doublings of each endpoint population relative to the GFP-tagged control strain it was competing against [72]. The relative competitive fitness of the endpoint populations was obtained by standardizing their competitive fitness to that of the rifampicin-sensitive ancestral strain within each set of competition experiments. Three biological and three technical replicates of each strain were assayed for each endpoint population.

To demonstrate that *lasR* mutations are beneficial under the same conditions of the selection experiment, the competitive fitness of three evolved rifampicin-resistant isolates that carried different *lasR* mutations and the initial *rpoB* mutant strains was determined relative to the rifampicin-sensitive ancestral strain using a modified version of the competition assays, in which all strains were regrown to exponential phase and co-cultured overnight with the GFP-tagged control strain in 5 ml Falcon tubes (BD Biosciences) using exactly the same growth conditions as those under which the selection experiment was carried out.

### (c) Whole-genome sequencing

The initial *rpoB* mutants and the endpoint populations of the selection experiment were streaked on M9KB agar plates and incubated overnight at 30°C. On the next day, three colonies of each population were randomly selected to inoculate M9KB liquid medium incubated at 30°C with shaking overnight. Genomic DNA was extracted using the DNeasy Blood and Tissue Kit (Qiagen, The Netherlands) according to the manufacturer's protocol. The 12 initial isolates of the rifampicin-sensitive ancestral strain and the three randomly selected colonies from the 12 endpoint populations, which were evolved from these initial isolates during the control experiment, were prepared for whole-genome sequencing using the same procedures.

To search for evidence of clonal interference in *rpoB* mutant populations, six random isolates from the 18th and 24th days of the selection experiment were selected for whole-genome sequencing from four selected *rpoB* mutant populations (H531Y-A, Q518L-C, Q518R-B and Q518R-C) using the same procedures as described above. Three additional random isolates from the four endpoint populations were whole-genome sequenced to obtain a total of six isolates for each of the three time points (excluding an isolate from the endpoint population of Q518R-C, which was not successfully sequenced).

Paired-end whole-genome sequencing with read length of 100 bp was performed using the HiSeq 2000 sequencing system (Illumina, USA). The average coverage was 44.1× (median coverage: 43.5×). Variants were identified using an experimentally validated in-house pipeline [72,73]. The initial *rpoB* mutant isolates were found to have the same genetic background, with the exception of the specified mutations in *rpoB*. We discarded mutations that were already present in all the initial isolates with respect to the *P. aeruginosa* PAO1 reference genome, analysing only those mutations that accumulated throughout the selection experiment.

### (d) Statistics

All statistical analyses were performed using JMP software v. 11 (SAS, USA). Unless otherwise stated, all statistical tests are two-tailed, and the level of significance is 0.05. The degrees of freedom are reported as subscripts next to the test statistics.

## Supplementary Material

Table 1

## Supplementary Material

Table 2

## Supplementary Material

Figure S1

## Data accessibility

Whole-genome sequencing data are available at the European Nucleotide Archive: http://www.ebi.ac.uk/ena/data/view/PRJEB11978.
