# Supplementary figures and images for: The genomic basis of adaptation to the fitness cost of rifampicin resistance in Pseudomonas aeruginosa
Source: Proc Biol Sci. 2016 Jan 13;283(1822):20152452. doi: 10.1098/rspb.2015.2452 (PMC4721101; doi:10.1098/rspb.2015.2452)

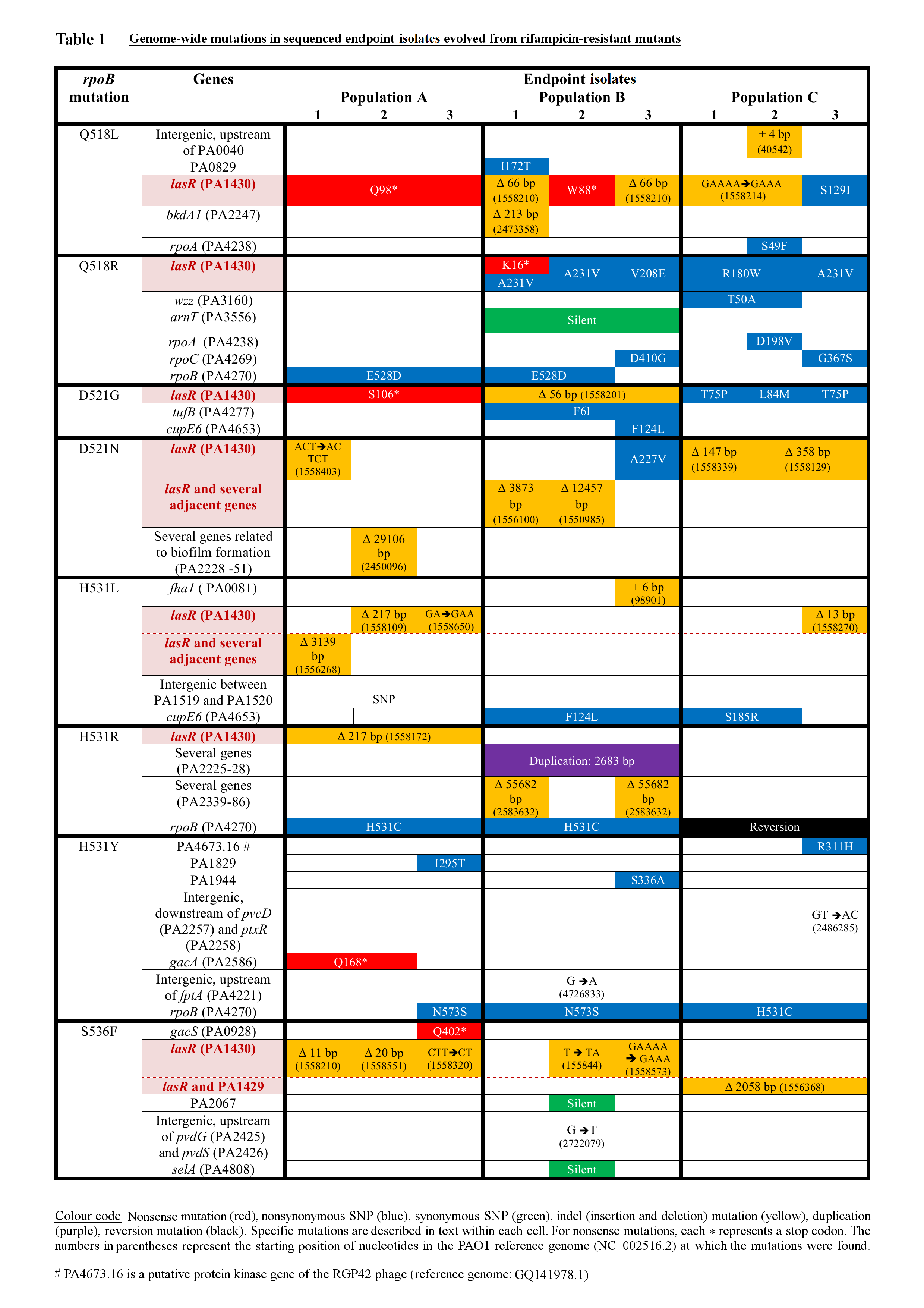

Supplement: Table 1 [file rspb20152452supp1.tif]

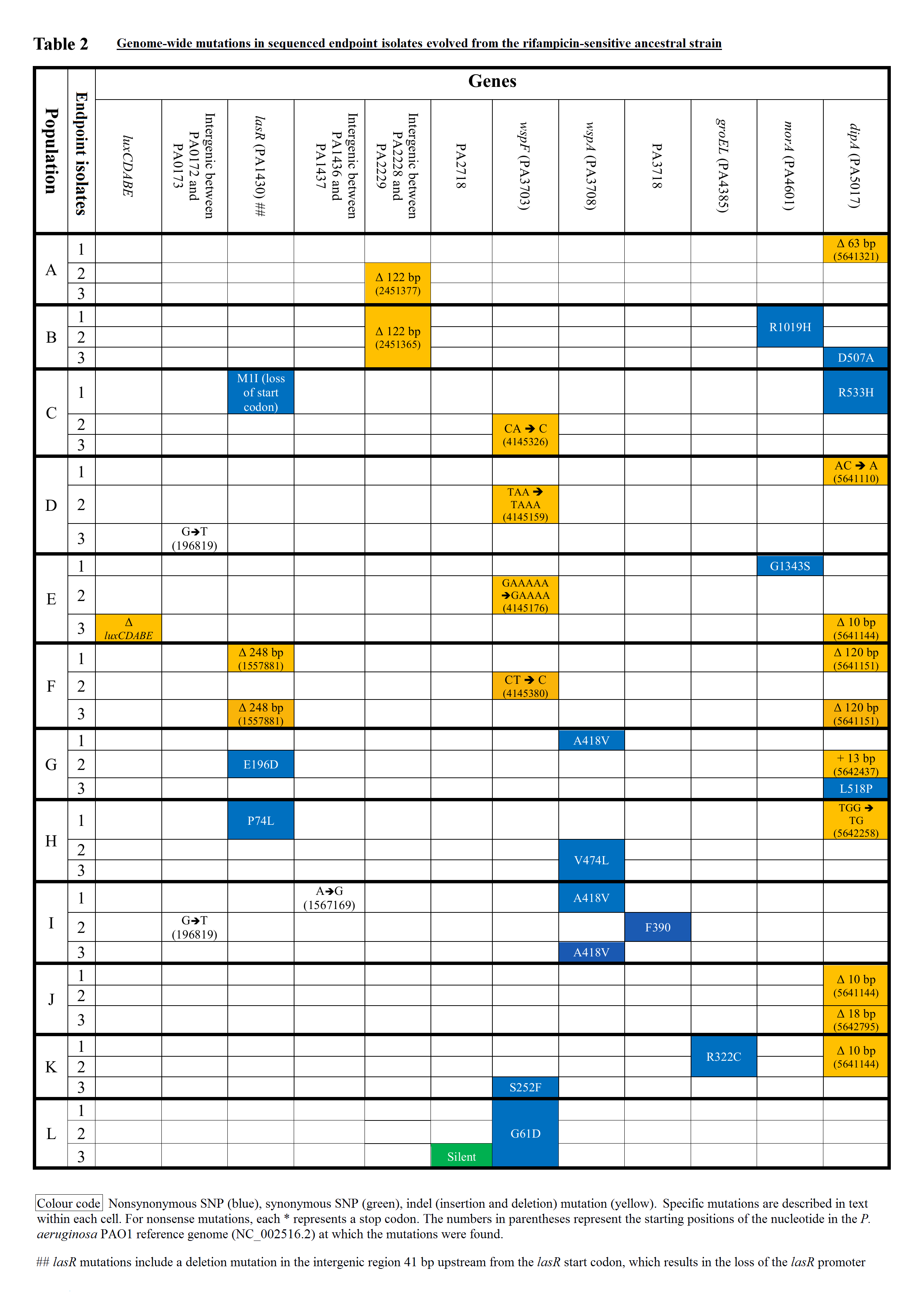

Supplement: Table 2 [file rspb20152452supp2.tif]
