# Supplementary material for: The genomic basis of adaptation to the fitness cost of rifampicin resistance in Pseudomonas aeruginosa
Source: Proc Biol Sci. 2016 Jan 13;283(1822):20152452. doi: 10.1098/rspb.2015.2452 (PMC4721101; doi:10.1098/rspb.2015.2452)
Supplement: Figure S1 [file rspb20152452supp3.pdf]

Figure S1, Electronic Supplementary Material

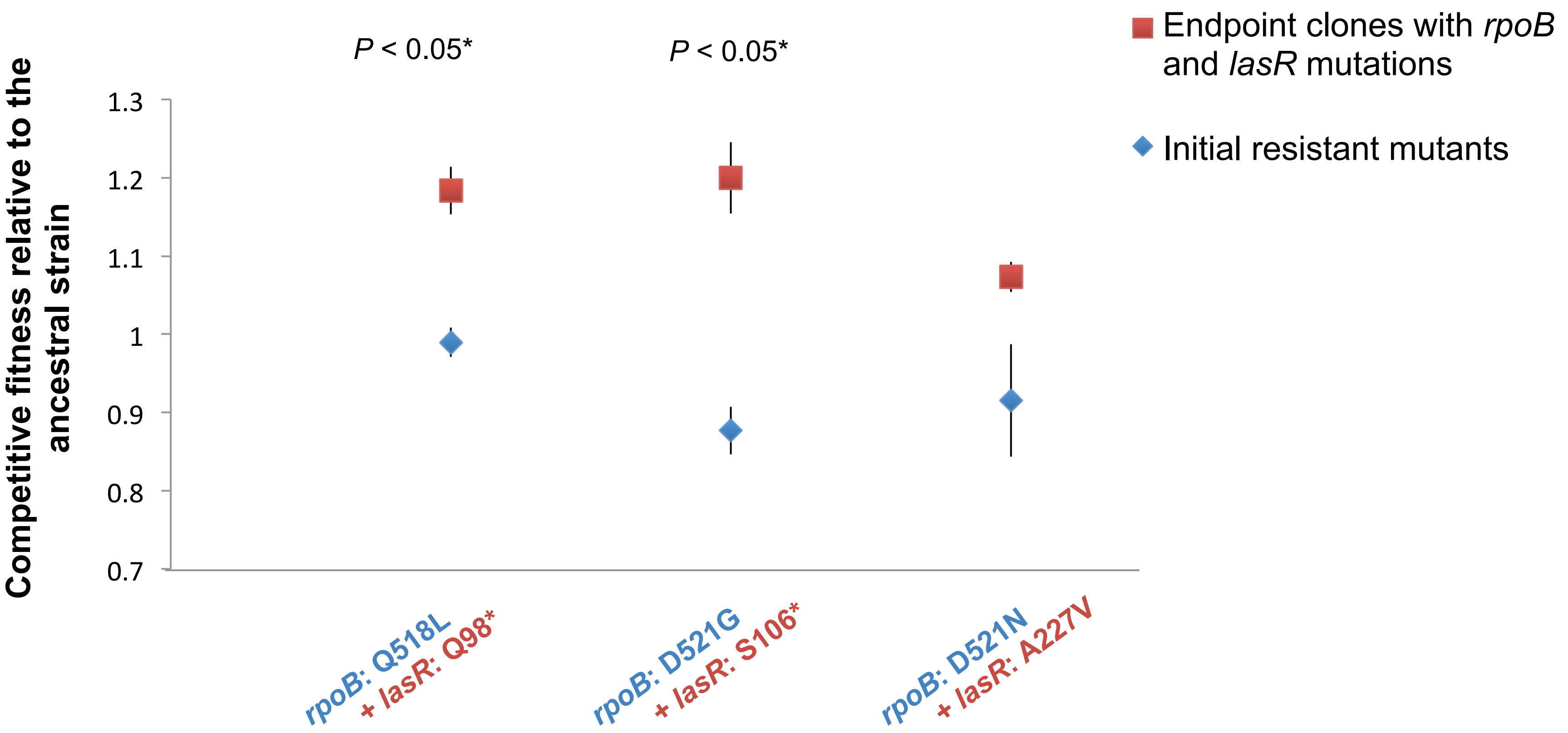

Figure S1: *lasR* mutations are beneficial.

The competitive fitness (mean ± s.e; n=3) of three initial *rpoB* mutants (blue diamonds) and three evolved endpoint clones (red squares) with single point mutations in the *lasR* gene were determined relative to the rifampicin-sensitive ancestral strain. Stop-gain mutations in *lasR* are denoted by the * symbol. All three evolved endpoint clones showed an increase in average competitive fitness compared to their ancestral *rpoB* mutants, two of which are statistically significant (two-sample t-test, $P < 0.05$).
